# A syrup containing l-arabinose and d-xylose appears superior to PEG-4000 as a bowel cleansing agent

**DOI:** 10.1186/s13568-024-01715-2

**Published:** 2024-06-01

**Authors:** Dezhi Wang, Xingchen Liao, Heng Zhang, Yilin Wang, Mingjie Zhang, Fangli Ren, Xianzong Ma, Jianqiu Sheng, Peng Jin, Dongliang Yu, Hui Xie, Xin Wang

**Affiliations:** 1 Department of Gastroenterology, The Seventh Medical Center of PLA General Hospital, #5 Nanmencang, Dongcheng District, Beijing, 100700 China; 2 Medical School of Chinese PLA, Chinese PLA General Hospital, Beijing, 100853 China; 3 State Key Laboratory of Membrane Biology, School of Medicine, Tsinghua University, Beijing, 100084 China

**Keywords:** Bowel cleansing, l-Arabinose, d-Xylose, Xylo-oligosaccharide (XOS), Polyethylene glycol-4000 (PEG-4000), Intestinal microbiota

## Abstract

Adequate bowel cleansing is crucial for endoscopic diagnosis and treatment, and the recovery of gut microbiota after intestinal cleansing is also important. A hypertonic syrup predominantly comprising l-arabinose and d-xylose (20% xylo-oligosaccharides) can be extracted from the hemicellulose of corn husks and cobs. l-Arabinose and xylo-oligosaccharides have been reported to relieve constipation and improve the gut microbial environment. This study evaluated the bowel cleansing effect of the aforementioned syrup and its influence on the organism and intestinal microbiota after cleansing in comparison with polyethylene glycol-4000 (PEG-4000) in mice. Bowel cleansing was performed using syrup or PEG-4000 in C57BL/6J mice, and the effect of intestinal preparation and its influence on serum electrolytes and gut microbiota after bowel cleansing were evaluated. The volume of intestinal residual feces in the syrup group was significantly lower than that in the PEG-4000 group. Additionally, syrup disturbed serum electrolytes more mildly than PEG-4000. Alpha diversity in the gut microbiota was significantly higher in the syrup group than in the PEG-4000 group on the first day after bowel cleansing. However, no difference in beta diversity was observed between the two groups. Syrup increased the abundance of *Bifidobacteria* and *Christensenella* and decreased the abundance of *Akkermansia* in comparison with PEG-4000 on the first day after bowel cleansing. Thus, this syrup has potential clinical use as a bowel cleansing agent given the above effects, its benefits and safety, and better taste and acceptability.

**Supplementary Information:**

The online version contains supplementary material available at 10.1186/s13568-024-01715-2.

## Introduction

Endoscopy is the gold standard for diagnosing various intestinal diseases and is an important therapeutic approach (Rastogi and Wani 2017). Adequate bowel cleansing is critical for high-quality and diagnostic endoscopy. The diagnostic performance, procedure time, cost price, and complication rate of endoscopy are directly related to proper bowel cleansing. Bowel cleansing agents are categorized as isotonic or hyperosmotic. The iso-osmotic bowel cleansing agent polyethylene glycol-4000 (PEG-4000) causes diarrhea by increasing the local osmotic pressure, which generates mechanical movement of water ions to clean the intestinal tract, achieving a good bowel cleansing effect. Furthermore, PEG-4000 is widely used in clinical practice because of its good reliability and safety (Di Nardo et al. 2014; Chen et al. 2013; Abe et al. 2021). However, PEG-4000 has a poor taste and requires a large intake of liquid, which may cause discomfort to patients, including nausea, vomiting, bloating, and electrolyte and intestinal microbiota disorders (Nyberg et al. 2010; Yoshioka et al. 2017; Chaussade and Minić 2003). New bowel cleansing agents need to be developed with improved tolerability and palatability to reduce the discomfort of bowel cleansing, improve patient compliance, and achieve better bowel cleansing scores.

The syrup produced by extracting hemicellulose from the cell wall of the corn plant through hydrolysis, purification, and crystallization is hyperosmotic and mainly comprises l-arabinose and d-xylose. l-Arabinose and xylo-oligosaccharide (XOS) relieve constipation (Tateyama et al. 2005; Jiang 2020; Song et al. 2023). l-Arabinose is not absorbed in the small intestine, inhibiting the metabolism and absorption of sucrose, reducing obesity and high blood sugar. l-Arabinose also promotes the proliferation of intestinal *Bifidobacteria* (Osaki et al. 2001; Tomioka et al. 2022; Tamura et al. 2012). Similarly, XOS in d-xylose is not readily degraded by digestive tract enzymes; thus, it can directly enter the large intestine and is preferentially used by *Bifidobacteria*, promoting their proliferation. *Bifidobacteria* subsequently further use XOS and generate large quantities of short-chain fatty acids, stimulate intestinal peristalsis, increase fecal wetness, and maintain the osmotic pressure, preventing constipation (Christensen et al. 2014; Mäkeläinen et al. 2010).

Therefore, the aforementioned syrup can be used as a high-osmolality bowel cleansing agent. Given that the sugar it contains is barely absorbed or digested in the intestine, no major fluctuations occur in the recipient’s blood glucose level (Juhász et al. 2020; Pasmans et al. 2022). Moreover, the syrup may have a beneficial effect on the intestinal microbiota, potentially aiding intestinal microbiota recovery after bowel cleansing. Our study aimed to evaluate the bowel cleansing effect of this syrup in comparison with control PEG-4000 in mice and its influence on the organism and intestinal microbiota after cleansing.

## Materials and methods

### Drugs

PEG-4000 (cat. #3,221,104) was purchased from Staidson Biopharmaceuticals Co., Ltd., Beijing, China. The syrup was purchased from Yingzhihao Biotechnology Co., Liaoning, China (Chen 2011, Chen 2012; Chen et al. 2013). The food production license number and product standard code of the syrup in China are SC10637148204742 and GB7101, respectively.

### Animals

Six-week-old C57BL/6 J mice (male and female, 18–22 g) were purchased from Charles River Laboratories (Beijing, China). Mice were housed at the Tsinghua Animal Facility in specific pathogen-free conditions with a 12 h dark/light cycle and free access to food and water. Male and female mice were kept in separate cages. Mice were adapted to the housing conditions for 7 days before the experiments were conducted. The experimental protocols were approved by the Animal Care and Use Committee of Tsinghua University (Protocol No. 21-CZJ1).

### Bowel preparation, blood index, and intestinal histopathological changes after bowel cleansing

Mice were randomly allocated to the following groups: control (normal diet and water), fasting (fasted for 16 h), syrup (five gavages with 250 μL of 20% syrup (v/v) at 30-min intervals, repeated after fasting for 12 h), and PEG-4000 (five gavages with 250 μL of PEG-4000 (A, 2.625 g/L; B, 2.879 g/L) at 30-min intervals, repeated after fasting for 12 h). Food (but not water) was withdrawn from the mice in the latter three groups 1 h before the first bowel cleansing. Mice were euthanized 1 h after cleansing ended, and the small and large intestines were removed. The residual feces from the intestines were collected and weighed. The harvested ileums and colons were stained with hematoxylin and eosin (H&E) (Cardiff et al. 2014). Blood samples were collected (Liu et al. 2019) and analyzed for blood urea nitrogen (BUN), glucose, phosphorus, magnesium, sodium, potassium, chlorine, calcium, and plasma osmotic pressure by fully automatic biochemistry analyzer (ZY-1200, Kehua Bio-engineering Co., Ltd., Shanghai, China). The experiments were conducted in two rounds. The first round aimed to evaluate the bowel cleansing effect of the syrup, and to assess whether the syrup would damage the intestinal mucosa. Each group consisted of 3 males and 3 females, housed in separate cages for each gender. It should be noted that, in this round, blood was not collected from the mice since the ileum needed to be isolated immediately to avoid damage of digestive enzymes to the small intestinal mucosal barrier due to small intestinal ischemia after euthanasia. The second round aimed to analyze the potential adverse effects of blood electrolyte disturbance after applying syrup for bowel cleansing. Mice were grouped according to the above treatments (1 cage with 3 males and 1 cage with 3 females in the control group, 1 cage with 3 males and 1 cage with 3 females in the fasting group, 1 cage with 4 males and 1 cage with 4 females in the syrup group, 1 cage with 4 males and 1 cage with 4 females in the PEG-4000 group). In this round, the bowel cleansing effect was also evaluated after collecting the blood.

### 16S rRNA gene sequencing

The mice were divided into the syrup (n = 6, 1 cage with 3 males and 1 cage with 3 females) and PEG-4000 groups (n = 6, 1 cage with 3 males and 1 cage with 3 females). Bowel cleansing was performed as described earlier. Freshly excreted feces were collected from the mice before bowel cleansing (defined as day 0) and on days 1 and 7 after bowel cleansing. DNA was extracted and stored at − 20 °C prior to further analysis. PCR amplification of the bacterial 16S rRNA gene V3–V4 region was performed using the forward primer (5'-ACTCCTACGGGAGGCAGCA-3') and the reverse primer (5'-GGACTACHVGGGTWTCTAAT-3'). Thermal cycling consisted of initial denaturation at 98 °C for 2 min, followed by 25 cycles consisting of denaturation at 98 °C for 15 s, annealing at 55 °C for 30 s, and extension at 72 °C for 30 s, with a final extension of 5 min at 72 °C. Sequence data analyses were mainly performed using the QIIME2 (Rai et al. 2021) and R packages (v3.2.0) (Olson et al. 2019). Operational taxonomic units (OTUs) were defined at 97% sequence similarity. The ribosomal database project classifier was applied to systematically classify OTU sequences with reference to the Silva database (Quast et al. 2013). The raw sequencing data were deposited in the NCBI Sequence Read Archive database under accession number PRJNA1056507.

### Chromatography$-$mass spectrometry (GC$-$MS) and high-performance liquid chromatography (HPLC) analyses

Syrup sugar was analyzed using GC$-$MS. The samples were prepared and extracted after being crushed, diluted, vortexed, ultrasonicated, and evaporated. Then, the evaporated sample was transferred to the lyophilizer for freeze-drying. The residue was used for further derivatization. The mass spectral peak intensity data of the corresponding quantitative signals of each concentration of the standards were obtained according to the different concentrations of the standard solutions of the 32 targeted sugars. Subsequently, the standard curves of different substances were plotted with the concentration ratio as the horizontal coordinate and the area ratio as the vertical coordinate. The integrated peak area ratios of all the samples were substituted into the linear equation of the standard curve to calculate the actual content. XOS in d-xylose was determined using HPLC analyses. The standard and sample solutions were added to the liquid chromatograph, and the corresponding peak times were recorded. XOS was quantified according to the peak areas.

### Statistical analyses

The difference between the two groups was compared using Student’s *t* test. The data were evaluated by one-way analysis of variance (ANOVA) followed by Tukey’s post hoc test for three or more groups. All analyses were performed using GraphPad Prism 9.4 (GraphPad Software Inc., San Diego, CA, USA).

## Results

### Main components of the syrup are l-arabinose and d-xylose

**Fig. 1:**
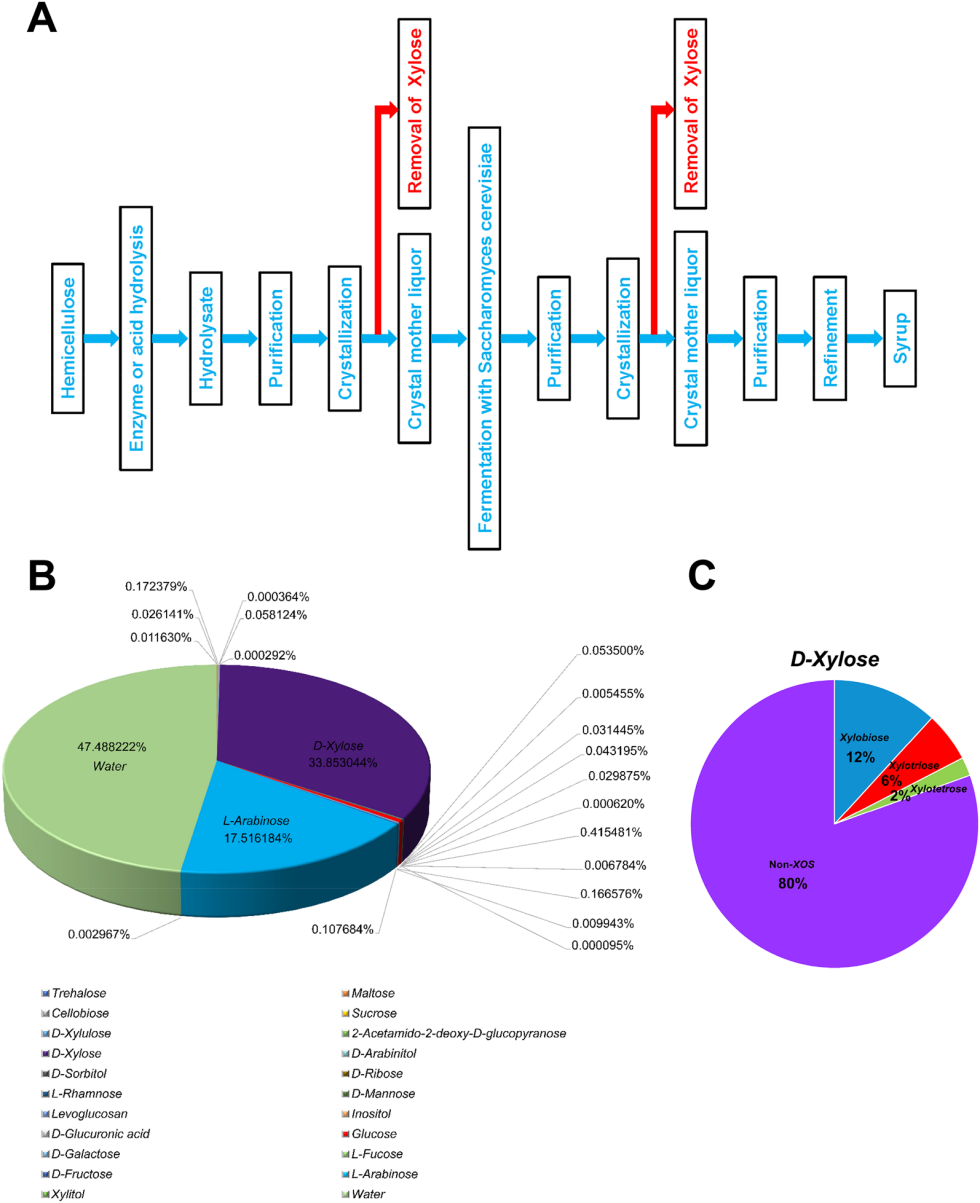
Main components of the syrup are l-arabinose and d-xylose. **A** Flowchart of the syrup production process. **B** Analysis of the composition of syrups by chromatography$-$mass spectrometry (GC$-$MS). **C** Analysis of xylo-oligosaccharide (XOS) in d-xylose by high-performance liquid chromatography (HPLC)

The manufacturing process (Fig. 1A) produced the syrup from hemicellulose after hydrolysis, purification, crystallization, and refinement. Mass spectrometry analysis was performed to identify the composition and content of the syrup. In addition, 21 sugar types were identified in the syrup through a GC/MS analysis of 32 sugars (Fig. 1B, Additional file 1: Table S1). A pie chart was generated to depict the types and levels of the sugars in the syrup (Fig. 1B); except for water, the two predominant syrup components were d-xylose and l-arabinose, accounting for 33% and 17%, respectively. We then further measured the syrup d-xylose XOS content, revealing xylobiose, xylotriose, and xylotetrose concentrations of 12%, 6%, and 2%, respectively (Fig. 1C).

### Syrup exhibits a more pronounced bowel cleansing effect than PEG-4000

**Fig. 2:**
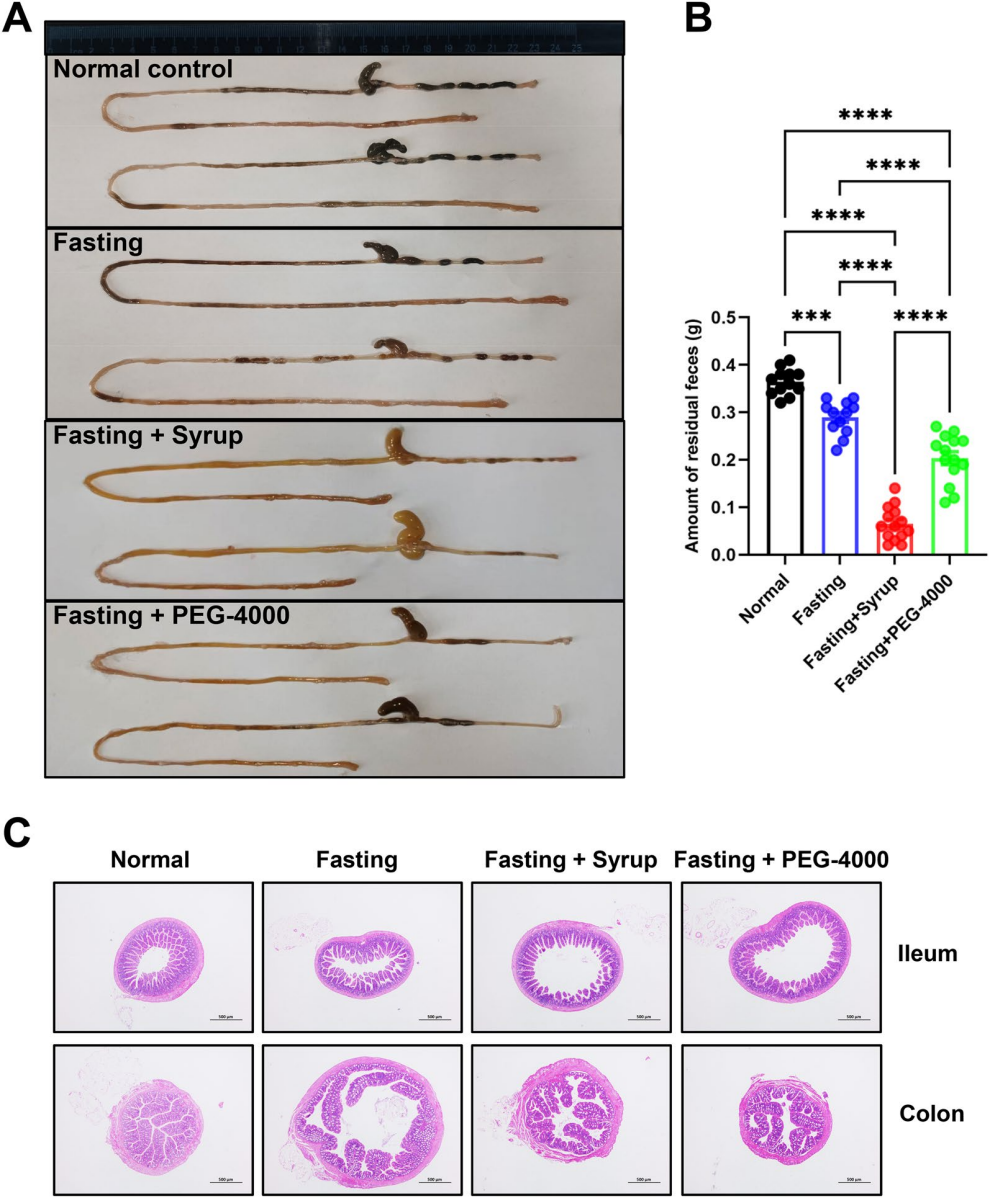
Syrup exhibits a more pronounced bowel cleansing effect than polyethylene glycol-4000 (PEG-4000). Mice were randomly allocated to the following groups: control (n = 12; administered a normal diet and water), fasting (n = 12; fasted for 16 h), syrup (n = 14; five gavages with 250 μL of 20% syrup (v/v) at 30-min intervals, repeated after fasting for 12 h), and PEG-4000 (n = 14; five gavages with 250 μL of PEG-4000 (A, 2.625 g/L; B, 2.879 g/L) at 30-min intervals, repeated after fasting for 12 h). Food (but not water) was withdrawn from mice in the latter three groups 1 h before bowel cleansing. Mice were euthanized 1 h after the end of cleansing. **A** Entire small and large intestines. **B** Volume of residual feces in intestines. **C** Hematoxylin and eosin (H&E) staining of small and large intestinal tissues (40×). Data are expressed as mean ± standard error of the mean (SEM). ^*^P < 0.05, ^**^P < 0.01, ^***^P < 0.001, and ^****^P < 0.0001

The small and large intestines of the mice were removed, and the residual intestinal feces were collected and weighed to determine the bowel cleansing effect of the syrup. Compared with the control and fasting groups, PEG-4000 and syrup gavages more effectively removed the intestinal feces and achieved a qualified bowel cleansing effect; the volume of intestinal residual feces in the syrup group was also significantly lower than that in the PEG-4000 group, indicating that the syrup showed a better bowel cleansing effect (Fig. 2A, B). Meanwhile, ileal and colon H&E staining revealed no abnormalities in the intestinal tracts cleansed with PEG-4000 or the syrup (Fig. 2C).

### Syrup bowel cleansing resulted in milder serum electrolyte disturbance than PEG-4000

**Fig. 3:**
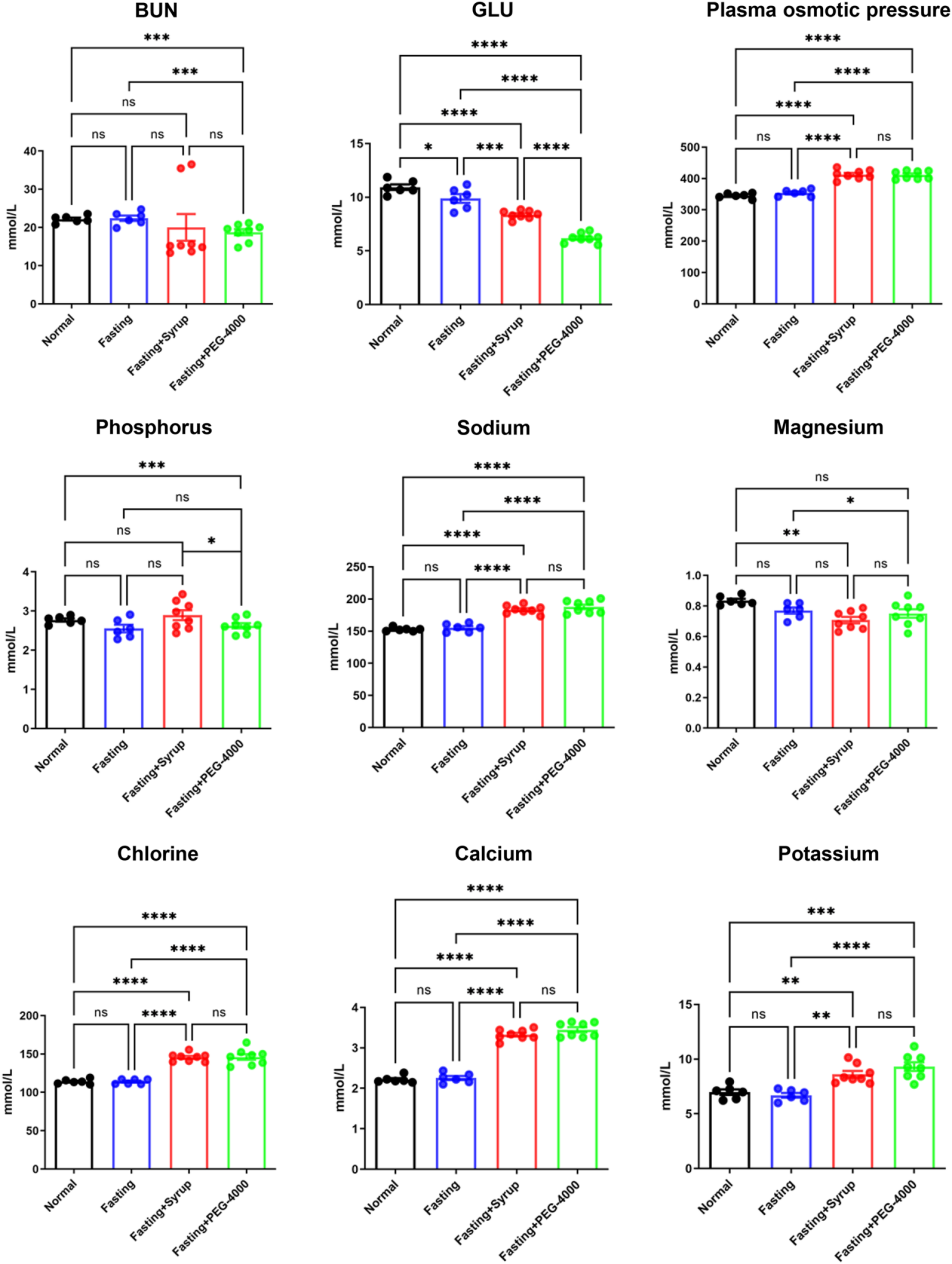
Syrup bowel cleansing resulted in milder serum electrolyte disturbance than polyethylene glycol-4000 (PEG-4000). Mice were randomly divided into the control (n = 6), fasting (n = 6), syrup (n = 8), and PEG-4000 groups (n = 8). The levels of serum electrolytes and plasma osmotic pressure are shown. Data are expressed as mean ± standard error of the mean (SEM). ^*^P < 0.05, ^**^P < 0.01, ^***^P < 0.001, and ^****^P < 0.0001

Blood plasma osmolality and electrolyte levels were examined to assess the potential adverse effects of blood electrolyte disturbance after applying syrup for bowel cleansing. No significant differences in plasma osmolality or BUN, sodium, magnesium, chloride, calcium, and potassium concentrations were observed in mice in the syrup group versus those in the PEG-4000 group (Fig. 3). However, notably, the serum concentrations of glucose and phosphorus were relatively closer to normal standards in the syrup group versus those in the PEG-4000 group.

### Syrup bowel cleansing affected the intestinal microbiota community composition less than PEG-4000

**Fig. 4:**
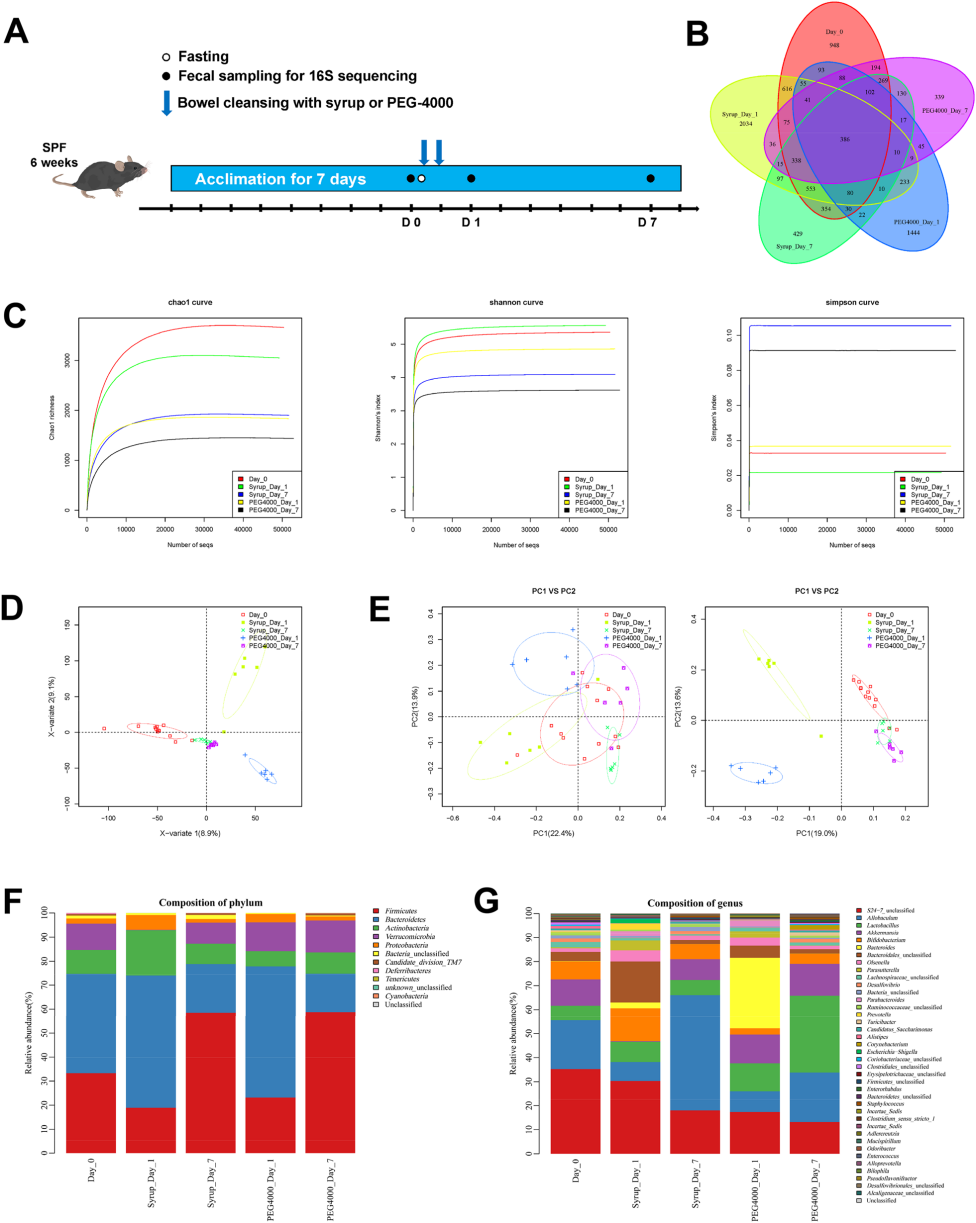
Syrup bowel cleansing affected the intestinal microbiota community composition less than polyethylene glycol-4000 (PEG-4000). Freshly excreted feces were collected from mice in the syrup group and PEG-4000 group before bowel cleansing and on days 1 and 7 after bowel cleansing for 16S rRNA gene sequencing (n = 6 per group). **A** Flowchart of the mouse fecal 16S rRNA experiment. **B**, **C** The intestinal microbiota α diversity after using PEG-4000 and syrup for bowel cleansing. **D**, **E** The intestinal microbiota β diversity after using PEG-4000 and syrup for bowel cleansing. **F**, **G** The relative abundance of intestinal microbiota community composition at the phylum and genus levels

Freshly excreted feces were collected from mice in the syrup and PEG-4000 groups before bowel cleansing and on days 1 and 7 after bowel cleansing for 16S rRNA gene sequencing to explore intestinal microbiota changes (Fig. 4A). First, the microbiota alpha diversity at the OTU level was analyzed by Chao1 richness, Shannon curves, and Simpson curves and was significantly higher in the syrup group than in the PEG-4000 group on the first day after bowel cleansing (Fig. 4C). Microbiota beta diversity analysis was then performed (Fig. 4B, D, E). The Venn diagram shows the distribution of OTUs across different groups, where the numbers represent specific or shared OTUs, the overlapping areas indicate OTUs shared by different groups, and the nonoverlapping areas indicate OTUs specific to different groups (Fig. 4B). Partial least squares discrimination analysis showed good model fit (Fig. 4E), and the weighted and unweighted principal coordinate analysis (PCoA) showed that the difference in OTUs between the PEG-4000 and syrup groups was large on day 1 compared with that on day 0. However, this difference recovered on day 7 to a certain extent.

### Changes in intestinal microbiota at the phylum and genus level after bowel cleansing with PEG-4000 or syrup

**Fig. 5:**
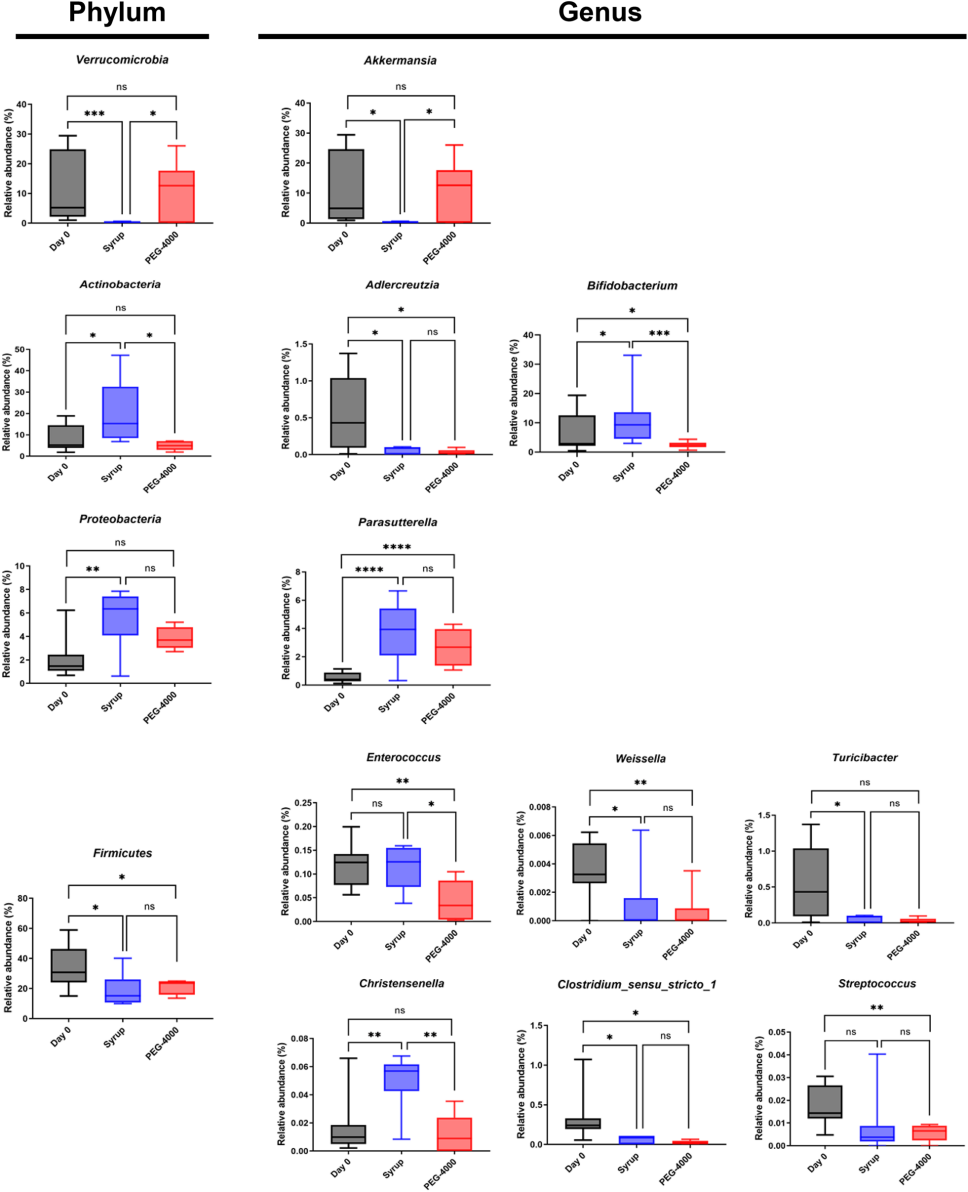
Changes in intestinal microbiota at the phylum and genus levels after using polyethylene glycol-4000 (PEG-4000) and syrup for bowel cleansing. The changes in intestinal microbiota on the first day after bowel cleansing were shown. Data are expressed as mean ± standard error of the mean (SEM). ^*^P < 0.05, ^**^P < 0.01, ^***^P < 0.001, and ^****^P < 0.0001

Finally, the community composition of the five groups was determined in terms of phylum (Fig. 4F) and genus (Fig. 4G), and the difference was further quantitatively analyzed (Fig. 5). Significant differences in community compositions were observed among the day 0 group, syrup day 1 group, and PEG-4000 day 1 group at the phylum and genus levels (Fig. 5). Compared with the day 0 group, the abundance of *Verrucomicrobia* and *Firmicutes* in the syrup day 1 group was significantly decreased, while that of *Actinobacteria* and *Proteobacteria* were increased. The decreased abundance of *Verrucomicrobia* was mainly caused by a decrease in *Akkermansia* abundance. The increased abundance of *Actinobacteria* and *Proteobacteria* was mainly caused by an increase in *Bifidobacteria* and *Parasutterella* abundance, respectively. Regarding *Firmicutes*, there was a significant decrease in the abundance of *Weissella*, *Turicibacter*, *Clostridium *sensu stricto* 1*, and *Streptococcus*. In addition, in comparison with that in the PEG-4000 day 1 group, in the syrup day 1 group, the abundance of *Akkermansia* was significantly decreased, but the abundance of *Bifidobacteria*, *Christensenella*, and *Enterococcus* was significantly elevated. Notably, no significant difference was observed among the day 0 group, syrup day 7 group, and PEG-4000 day 7 group at the phylum level (data not shown), indicating intestinal microbiota gradually recovered within a week after bowel cleansing.

## Discussion

The most representative physiological effect of l-arabinose is that it selectively affects the sucrase enzyme in the small intestine, thus inhibiting the absorption of sucrose (Krog-Mikkelsen et al. 2011). Given that this sugar is also not readily completely absorbed by the small intestine, many researchers believe that it can inhibit metabolic diseases, such as obesity and type 2 diabetes (Osaki et al. 2001; Hao et al. 2015; Tomioka et al. 2022). l-Arabinose can also promote organic acid and short-chain fatty acid synthesis, building an acidic intestinal environment that promotes *Bifidobacteria* proliferation. Our study revealed that compared with that in the day 0 group, the abundance of *Bifidobacteria* was significantly increased in the syrup day 1 group, consistent with previous findings (Li et al. 2023; Tamura et al. 2012; Hao et al. 2015). In addition, l-arabinose regulates the composition, abundance, and diversity of the intestinal microbiota and inhibits colitis in mice (Li et al. 2019; Zhao et al. 2019). l-Arabinose significantly upregulates the phosphorylation levels of 5' adenosine monophosphate-activated protein kinase (AMPK) and its downstream protein acetyl-CoA carboxylase (ACC), thus inhibiting hepatic gluconeogenesis and regulating hyperglycemia in mice fed a high-fat and high-sugar diet (Wang et al. 2021). l-Arabinose also exerts anti-inflammatory effects and protects the gastrointestinal mucosal barrier by decreasing M1 macrophage polarization (Kang et al. 2023). XOS, a functional polymerized sugar consisting of two to seven xylose molecules bound by β-1,4 glycosidic bonds, has demonstrated various effects on human health, such as inducing immunomodulation, antitumor, and antioxidant activities (Chen et al. 2021a, b). In addition, XOS regulates intestinal microbiota and promotes *Bifidobacteria* proliferation. XOS can selectively promote the proliferation of beneficial bacteria, such as *Bifidobacterium intestinalis (B. intestinalis)*, leading them to become the dominant intestinal flora; XOS can also regulate the intestinal microecological balance and promote intestinal health (Mäkeläinen et al. 2010; Precup et al. 2022). *Bifidobacterium bifidum (B. bifidum)* is a normal inhabitant of the human intestinal tract and can selectively metabolize XOS or xylose, producing large quantities of short-chain fatty acids; in contrast, *Staphylococcus*, *Escherichia coli (E. coli),* and many *Clostridia* in the intestinal tract cannot use XOS or xylose (Johnson et al. 2006). *Bifidobacteria* metabolize XOS, producing short-chain fatty acids, mainly lactic acid and acetic acid, which can lower the intestinal tract pH and inhibit the proliferation of other harmful bacteria, reducing harmful metabolic substances in the body, such as indole, phenol, ammonia, and cadaverine. This effect reduces the risk of developing colon cancer and other diseases (Arora et al. 2013; Pang et al. 2021; Ebersbach et al. 2012). Improving the acidic environment of the intestinal tract can also stimulate intestinal peristalsis, increase fecal wetness, and promote defecation, thus preventing constipation and reducing the hazards of persistent stools in constipated patients (Childs et al. 2014). d-xylose has been reported to selectively inhibit sucrase activity in a noncompetitive manner (Asano et al. 1996). When consumed with glucose solutions or high carbohydrates, d-xylose lowers serum glucose levels in subjects within 30 min (Jun et al. 2016), and the glucose-inhibiting effect of d-xylose may occur in part through sucrase inhibition as well as through stimulation of glucagon-like peptide-1 secretion (Wu et al. 2013).

Electrolyte balance in the body is regulated by two main sources: oral intake and renal excretion. A supraphysiologic intake of large volumes of fluid during cleansing or a significant fluid loss via renal or intestinal routes may lead to electrolyte imbalance. In addition, all purgatives function via the osmotic effects of their ingredients, which may also contribute to disturbances in water and electrolyte balance (Schneider et al. 2022). This study revealed that the syrup assessed exhibits a more pronounced bowel cleansing effect than PEG-4000. In addition, compared with the mice in the PEG-4000 group, those in the syrup group did not show significant plasma osmolality or electrolyte disturbances. However, the syrup group showed better blood glucose and phosphorus levels than the PEG-4000 group. l-Arabinose and d-xylose, the main components of the syrup, can inhibit sucrase and thereby delay the digestion of sucrose. This may be the reason why the blood glucose concentration after bowel cleansing by using the syrup was closer to the standard level compared with PEG-4000.

The syrup did not cause greater alterations in the intestinal microbiota community composition abundance than PEG-4000, and the community composition abundance was substantially restored on day 7 after bowel cleansing. Interestingly, on the first day after bowel cleansing, compared with the PEG-4000 group, the syrup group showed an increase in the abundance of *Bifidobacteria* and *Christensenella* but a decrease in the abundance of *Akkermansia*. The syrup was determined to consist mainly of 47% water, 33% d-xylose, and 17% l-arabinose; the d-xylose contained 20% XOS. It is possible that the d-xylose, XOS, and l-arabinose components of the syrup caused the aforementioned differences in intestinal bacterial composition abundance. *Bifidobacteria* is an important beneficial intestinal microbiota with many important physiological functions in human health, such as biological barriers, nutritional effects, immune enhancement, improvement of gastrointestinal function, and anti-aging. Moreover, *B. bifidum* probiotic preparation has been widely used in the clinic (Hidalgo-Cantabrana et al. 2017; Tojo et al. 2014; Wei et al. 2018; Chen et al. 2021b, a). *Christensenellaceae*, widely found in the intestines of humans and animals, are important for host health, and are significantly and negatively associated with body mass index and metabolic disorders, such as fat deposition, inflammatory bowel disease (IBD), and metabolic syndrome (Waters and Ley 2019; Tavella et al. 2021). *Akkermansia*, a genus of Gram-negative, anaerobic bacterium in the phylum *Verrucomicrobia* and the family *Akkermansiaceae*, includes the species *Akkermansia muciniphila (A. muciniphila)*, which resides in the human intestinal tract and affects metabolism. Over the past decade, a growing number of studies have demonstrated a reduced abundance of *A. muciniphila* in patients with diabetes, cardiovascular disease, inflammatory bowel disease, or neurological disease and that increasing its abundance can help improve metabolic function in patients with diabetic and obese individuals (Cani et al. 2022; Zhai et al. 2019; Zhou and Zhang 2019; Rodrigues et al. 2022; Zheng et al. 2022). Interestingly, in this study, the abundance of *Bifidobacteria* and *Christensenellaceae* was significantly increased, but that of *Akkermansia* was significantly decreased after bowel cleansing with syrup. In addition, the syrup group showed better alpha diversity, potentially resulting from modulation of the composition, diversity, and abundance of the intestinal microbiota in response to l-arabinose (Behzadi Nia et al. 2020), compared with the PEG-4000 group. *Akkermansia* probiotic supplementation after bowel cleansing will be investigated in subsequent studies along with investigations into the syrup components that underlie the changes in *Christensenellaceae* and *Akkermansia* abundance.

Overall, the syrup demonstrated a better bowel cleansing effect than PEG-4000, increasing the abundance of beneficial intestinal bacteria, such as *Bifidobacteria* and *Christensenella*, on the first day after bowel cleansing. Reduced serum electrolyte disturbances were also observed, and no severe intestinal microbiota dysbiosis was present. The syrup has potential clinical use as a bowel cleansing agent, given its effects, benefits, cost-effectiveness, safety, palatability, and acceptability.

### Supplementary Information

Supplementary Material 1

## Data Availability

All data used to support the findings of this study are included in the article.
