# Supplementary material for: A syrup containing l-arabinose and d-xylose appears superior to PEG-4000 as a bowel cleansing agent
Source: AMB Express. 2024 Jun 1;14:63. doi: 10.1186/s13568-024-01715-2 (PMC11144180; doi:10.1186/s13568-024-01715-2)
Supplement: Supplementary file 1 — Supplementary Material 1 [file 13568_2024_1715_MOESM1_ESM.pdf]

Journal Name: AMB Express

Manuscript Title: A syrup containing L-arabinose and D-xylose appears superior to PEG-4000 as a bowel cleansing agent

The names of the authors: Dezhi Wang[1, 2#], Xingchen Liao[1, 2#], Heng Zhang[1, 2#], Yilin Wang[1, 2], Mingjie Zhang[1], Fangli Ren[3], Xianzong Ma[1, 2], Jianqiu Sheng[1, 2], Peng Jin[1], Dongliang Yu[1], Hui Xie[1]*, Xin Wang[1]*

[#]These authors contributed equally to this study.

Corresponding author: Hui Xie and Xin Wang*

The affiliations and addresses of the authors: [1]Department of Gastroenterology, the Seventh Medical Center of PLA General Hospital, Beijing 100700, China,

[2]Medical School of Chinese PLA, Chinese PLA General Hospital, Beijing 100853, China,

[3]State Key Laboratory of Membrane Biology, School of Medicine, Tsinghua University, Beijing 100084, China.

The e-mail address, telephone and fax numbers of the corresponding author: Hui Xie*, E-mail: xiehuixhnk@163.com, Tel: +86-10-66721014, Fax: +86-10-66721299; Xin Wang*, E-mail: chan805126@126.com, Tel: +86-10-66721014, Fax: +86-10-66721299.

Table S1. 21 sugar types were identified in the syrup through a GC/MS analysis of 32 sugars.

Index	Compounds	Class	Q1 (Da)	Molecular Weight	Ion mode	Formula	M_1-6-1	M_1-6-2	M_1-6-3	M_1-6-4	M_1-6-5	M_1-6-6	MWXS231122a_R1	MWXS231122a_R2	MWXS231122a_R3	cpd_ID	CAS
Tre	Trehalose	disaccharide	361	342.116215	Positive	C12H22O11	0.122639423	0.109108838	0.118318675	0.113517949	0.119837712	0.110675355	0.112945	0.1169208	0.122712	C01083	99-20-7
Lac	Lactose	disaccharide	319	342.116215	Positive	C12H22O11	N/A	N/A	N/A	N/A	N/A	N/A	N/A	N/A	N/A	C00243	63-42-3
Mal	Maltose	disaccharide	361	342.116215	Positive	C12H22O11	0.289788462	0.249815293	0.252935743	0.270254438	0.270526418	0.252299853	0.236232	0.258626	0.27217	C00208	69-79-4
Phe	Phenylglucoside	disaccharide	361	256.09469	Positive	C12H16O6	0.000271148077	N/A	N/A	N/A	N/A	0.0000233011269	0.0000758922	0.0000896376	0.0000636262	C11611	1464-44-4
Cel	Cellobiose	disaccharide	361	342.116215	Positive	C12H22O11	1.83322692	1.59765045	1.75584739	1.68756016	1.78192535	1.66239098	1.66759	1.733154	1.794802	C00185	528-50-7
Suc	Sucrose	disaccharide	437	342.116215	Positive	C12H22O11	0.00375930769	0.00248975174	0.00255222892	0.0028982643	0.00303555986	0.00255527683	0.00542278	0.0047577	0.0053037	C00089	57-50-1
Met	Methyl beta-D-galactopyranoside	monosaccharide	204	194.07904	Positive	C7H14O6	N/A	N/A	N/A	N/A	N/A	N/A	N/A	N/A	N/A	C03619	1824-94-8
Gal-A	D-Galacturonic acid	monosaccharide	333	194.042655	Positive	C6H10O7	N/A	N/A	N/A	N/A	N/A	N/A	N/A	N/A	N/A	C00333	685-73-4
Deo	Deoxyglucose	monosaccharide	319	164.068475	Positive	C6H12O5	N/A	N/A	N/A	N/A	N/A	N/A	N/A	N/A	N/A	C00586	154-17-6
1-5-Anh	1,5-Anhydroglucitol	monosaccharide	191	164.068475	Positive	C6H12O5	N/A	N/A	N/A	N/A	N/A	N/A	N/A	N/A	N/A	C07326	154-58-5
Ribono-1-4-lactone	D-Ribono-1,4-lactone	monosaccharide	117	148.037175	Positive	C5H8O5	N/A	N/A	N/A	N/A	N/A	N/A	N/A	N/A	N/A	-	5336-08-3
Man-6-pho	D-Mannose-6-phosphate sodium salt	monosaccharide	471	273.024971	Positive	C6H12NaO9P	N/A	N/A	N/A	N/A	N/A	N/A	N/A	N/A	N/A	-	70442-25-0
2-Deo-ribose	2-Deoxy-D-ribose	monosaccharide	307	134.05791	Positive	C5H10O4	N/A	N/A	N/A	N/A	N/A	N/A	N/A	N/A	N/A	C01801	533-67-5
Xylulose	D-Xylulose	monosaccharide	263	150.052825	Positive	C5H10O5	0.594842308	0.561654419	0.579413655	0.58633925	0.586949103	0.562310632	0.583216	0.590446	0.585998	C00310	551-84-8
2-Ace-2-Deo-D-Glucosamine	2-Acetamido-2-deoxy-D-glucopyranose	monosaccharide	319	221.089939	Positive	C8H15NO6	0.00290823077	0.0029706852	0.00327495984	0.00311220907	0.0030837809	0.00269471828	0.00277356	0.00271208	0.00277322	C00140	7512-17-6
Xylose	D-Xylose	monosaccharide	307	150.052825	Positive	C5H10O5	348.075	329.062562	340.188755	336.808679	342.355793	329.701127	339.866	339.666	341.05	C00181	58-86-6
Arabinitol	D-Arabinitol	monosaccharide	307	152.068475	Positive	C5H12O5	0.553790385	0.51233565	0.524658635	0.545635108	0.548003878	0.521340519	0.52687	0.538014	0.544388	C01904	488-82-4
Sorbitol	D-Sorbitol	monosaccharide	307	182.07904	Positive	C6H14O6	0.0577848077	0.0517271102	0.0519293173	0.0568319527	0.054635967	0.0534183244	0.0531968	0.0549144	0.0565104	C00794	50-70-4
Ribose	D-Ribose	monosaccharide	307	150.052825	Positive	C5H10O5	0.455046154	0.413543198	0.431985944	0.428315582	0.429905962	0.41522587	0.443698	0.436052	0.433794	C00121	50-69-1
Rha	L-Rhamnose	monosaccharide	321	164.068475	Positive	C6H12O5	0.331246154	0.3043714	0.309164659	0.320309665	0.320727096	0.304587947	0.30782	0.316094	0.31576	C00507	3615-41-6
Man	D-Mannose	monosaccharide	319	180.06339	Positive	C6H12O6	0.317248077	0.280790467	0.297287149	0.30586785	0.301452254	0.289899069	0.29679	0.298236	0.301194	C00159	3458-28-4
Lev	Levoglucosan	monosaccharide	333	162.052825	Positive	C6H10O5	0.006113	0.00581968222	0.00541064257	0.00520260355	0.00590625303	0.00590536012	0.00718482	0.0071971	0.0070464	C22350	498-07-7
Inositol	Inositol	monosaccharide	265	180.06339	Positive	C6H12O6	0.0688628846	0.0664671301	0.0685742972	0.0655706114	0.0685079981	0.0668133268	0.06741	0.0675172	0.0708234	C00137	87-89-8
Glucuronic-A	D-Glucuronic acid	monosaccharide	160	194.042655	Positive	C6H10O7	0.000838523077	0.000874301887	0.000759945783	0.000726838264	0.000785335919	0.00071300147	0.001355214	0.001236434	0.00124722	C16245	6556-12-3
Glu	Glucose	monosaccharide	319	180.06339	Positive	C6H12O6	4.37613462	3.9134856	4.10648594	4.22968442	4.18823073	4.02245958	4.13918	4.19932	4.21828	C00031	50-99-7
Gal	D-Galactose	monosaccharide	319	180.06339	Positive	C6H12O6	1.72246538	1.5920576	1.6587751	1.69098619	1.67784973	1.62078981	1.648334	1.67526	1.705306	C00124	59-23-4
Fuc	L-Fucose	monosaccharide	117	164.068475	Positive	C6H12O5	0.106558269	0.0924516385	0.0973186747	0.103475345	0.106892293	0.0959159236	0.0980486	0.098179	0.0960526	C01019	2438-80-4
Fru	D-Fructose	monosaccharide	307	180.06339	Positive	C6H12O6	1.11960769	1.05120953	1.07	1.08237081	1.08969656	1.06206565	1.045082	1.082192	1.08931	C10906	7660-25-5
L-Ara	L-Arabinose	monosaccharide	307	150.052825	Positive	C5H10O5	179.193462	169.211519	176.60502	174.58856	175.686282	168.831945	178.5234	176.7774	177.039	C00216	10323-20-3
Xylitol	Xylitol	monosaccharide	307	152.068475	Positive	C5H12O5	0.0312944231	0.0280564052	0.0297636546	0.0305094675	0.0298047504	0.02877805	0.0291934	0.0293814	0.030272	C00379	87-99-0
Ribose-5-pho-Ba	Barium D-ribose-5-phosphate	monosaccharide	315	228.003507	Positive	C5H9BaO8P	N/A	N/A	N/A	N/A	N/A	N/A	N/A	N/A	N/A	-	15673-79-7
Raffinose	Raffinose	trisaccharide	361	504.16904	Positive	C18H32O16	N/A	N/A	N/A	N/A	N/A	N/A	N/A	N/A	N/A	C00492	512-69-6
